# Artemisinin Analogues as Potent Inhibitors of In Vitro Hepatitis C Virus Replication

**DOI:** 10.1371/journal.pone.0081783

**Published:** 2013-12-11

**Authors:** Susan Obeid, Jo Alen, Van Hung Nguyen, Van Cuong Pham, Philip Meuleman, Christophe Pannecouque, Thanh Nguyen Le, Johan Neyts, Wim Dehaen, Jan Paeshuyse

**Affiliations:** 1 Rega Institute for Medical Research, KU Leuven, Leuven, Belgium; 2 Molecular Design and Synthesis, Department of Chemistry, KU Leuven, Leuven, Belgium; 3 Institute of Marine Biochemistry, Vietnam Academy of Science and Technology, Hanoi, Vietnam; 4 Department of Clinical Chemistry, Microbiology and Immunology, University Ghent, Ghent, Belgium; Scripps Research Institute, United States of America

## Abstract

We reported previously that Artemisinin (ART), a widely used anti-malarial drug, is an inhibitor of *in vitro* HCV subgenomic replicon replication. We here demonstrate that ART exerts its antiviral activity also in hepatoma cells infected with full length infectious HCV JFH-1. We identified a number of ART analogues that are up to 10-fold more potent and selective as *in vitro* inhibitors of HCV replication than ART. The iron donor Hemin only marginally potentiates the anti-HCV activity of ART in HCV-infected cultures. Carbon-centered radicals have been shown to be critical for the anti-malarial activity of ART. We demonstrate that carbon-centered radicals-trapping (the so-called TEMPO) compounds only marginally affect the anti-HCV activity of ART. This provides evidence that carbon-centered radicals are not the main effectors of the anti-HCV activity of the Artemisinin. ART and analogues may possibly exert their anti-HCV activity by the induction of reactive oxygen species (ROS). The combined anti-HCV activity of ART or its analogues with L-N-Acetylcysteine (L-NAC) [a molecule that inhibits ROS generation] was studied. L-NAC significantly reduced the *in vitro* anti-HCV activity of ART and derivatives. Taken together, the *in vitro* anti-HCV activity of ART and analogues can, at least in part, be explained by the induction of ROS; carbon-centered radicals may not be important in the anti-HCV effect of these molecules.

## Introduction

Worldwide, an estimated 180 million people are chronically infected with the hepatitis C virus (HCV) [1]. The current therapy consists of pegylated interferon α (peg-IFNα), Ribavirin (RBV) in combination with either the protease inhibitor (PI) Telaprevir or Boceprevir. This combination therapy has been reported to be effective in up to 79% of the treated patients infected with HCV [1], [2]. PIs and many of the selective inhibitors of HCV replication that target the viral genome (including most of those in advanced clinical development) select rapidly for drug-resistant variants [3]. Alternatively, host targeting antivirals, such as the cyclophilin-binding molecule Alisporivir, have a high barrier to resistance [4], [5].

Artemisinin (ART), a sesquiterpene lactone with an endoperoxide function isolated from the plant *Artemisia annua* L, is widely used as an anti-malarial drug [6]–[8]. The drug has also been reported to exert anti-bacterial, anti-inflammatory and anti-angiogenic activities [9]–[12]. However, because of its low solubility and poor oral bioavailability, its therapeutic efficacy is not optimal [11], [13]. To combat these hurdles, numerous ART analogues were synthesized and evaluated for their potential anti-microbial effect [14]. Interestingly, some of these compounds exhibited, *in vitro,* anti-herpes viruses, anti-human cytomegalovirus, anti-human immunodeficiency virus and anti-hepatitis B virus activity [15]–[19]. We reported earlier that ART inhibits *in vitro* HCV replicon replication at concentrations that have no effect on host cell growth [24].

Here we report on the discovery of ART analogues that are more potent and selective inhibitors of HCV replication than the parent compound and propose by which mechanism they may do so.

## Materials and Methods

### Compounds

**Figure 1:**
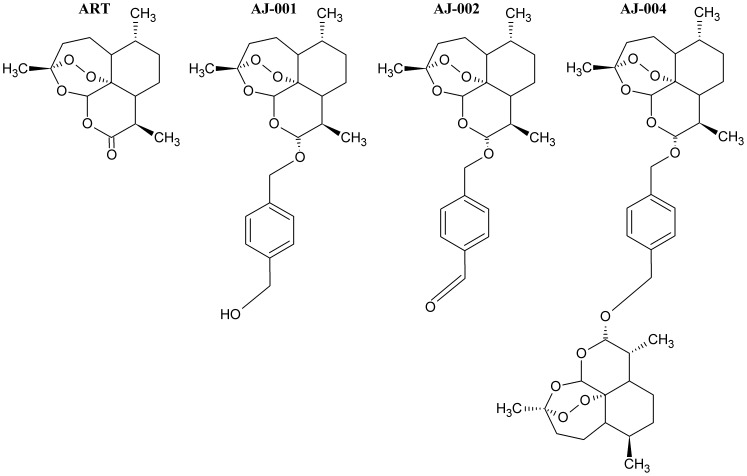
Structural formulae of Artemisinin and synthetic derivatives belonging to the first category AJ.

**Figure 2:**
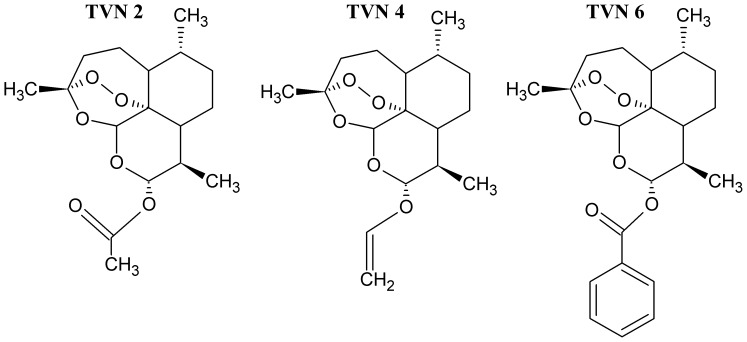
Structural formulae of Artemisinin and synthetic derivatives belonging to the second category TVN.

Artemisinin, Hemin and TEMPO compounds were purchased from Sigma (Bornem, Belgium). Artemisinin analogues (Fig. 1 and 2) were synthesized by methods that will be reported elsewhere [20].

### HCV Replicon Assay

Cells carrying HCV replicons I_389_luc-ubi-neo/NS3-3′/5.1 (Huh 5-2) were kindly provided by Prof. R. Bartenschlager (University of Heidelberg, Germany). Cells were cultured in Dulbecco’s modified Eagle’s Medium (DMEM, Gibco, Merelbeke, Belgium) supplemented with 10% heat-inactivated fetal bovine serum (Integro, Zaandam, The Netherlands), 1× non-essential amino acids, 100 IU/mL penicillin (Gibco), 100 µg/mL streptomycin (Gibco), and 250 µg/mL G418. Cell cultures were maintained at 37°C with 5% CO_2_.

### Antiviral Assay in HCV Replicon Cells

The antiviral assay was performed as described [21], [22]. Briefly, cells were seeded at a density of 5×10^3^ cells per well in 96-well cell culture plates in DMEM containing 250 µg/mL G418 at 37°C (5% CO_2_). After 24 hours of incubation, medium was replaced with fresh DMEM (without G418) and serial dilutions of the test compounds. Replicon RNA levels were determined by a quantitative reverse transcription polymerase chain reaction (qRT-PCR) or quantified by measuring the firefly luciferase activity in 96-well cell culture plates (Safire, Tecan, Austria).

### Antiviral Assay in the HCV Infectious System

The highly infectious HCV JFH-1/CS-N6 described by Delgrange et al [23] was used for the antiviral assays. A total of 7.2×10^3^ Huh 7.5.1 cells per well of a 96-well cell culture plate were incubated with the virus at specific infectivity of about 400 (400 HCV RNA copies per foci-forming unit [24]) and at the same time with serial dilutions of compounds. Following 3 days of incubation, medium was removed and cells were washed once and lysed to extract the intracellular RNA with the RNeasy kit (Qiagen). HCV RNA was quantified by means of qRT-PCR [25].

### qRT-PCR Assay

A qRT-PCR mixture contained: cellular RNA extract, HCV JFH-1 forward primer SF-JFH86 [5′-TGG CGT TAG TAT GAG TGT CGT ACA GCC TCC A-3′], reverse primer SR-JFH194 [5′-AAA GGA CCC AGT CTT CCC GGC AAT T-3′], and probe [5′-FAM-TGG TCT GCG GAA CCG GTG AGT ACA CC-TAMRA-3′], was performed at 50°C for 30 min, subsequent 15 min at 95°C and PCR amplification of 40 cycles of denaturation at 94°C for 20 s and annealing and extension at 60°C for 1 min in an ABI 7500 Taqman (Live Technologies).

### Cytostatic Assay

Cells were seeded at a density of 5×10^3^ or 7.2×10^3^ cells per well in a 96-well plate in complete DMEM in serial dilutions of the test compounds for Huh 5-2 and Huh 7.5.1 cells, respectively. After three days of incubation, cell viability was determined by MTS/PMS method (Promega). The 50% cytotoxic concentration (CC_50_) was defined as the concentration that inhibited the proliferation of exponentially growing cells by 50%.

### Drug Combination Studies

The effects of drug combinations were evaluated in a checker-board format using the method of Prichard and Shipman [26]. The theoretical additive effect was calculated from the dose–response curves of individual compounds by the equation Z = X+Y(1−X), where X represents the inhibition produced by first compound alone and Y the inhibition by the second compound alone. Z represents the effect produced by the combination of the first with the second compound. The theoretical additive surface is subtracted from the actual experimental surface, resulting in a horizontal surface that equals the zero plane when the combination is additive. A surface that lies above the zero plane indicates a synergistic effect of the combination, and a surface below the zero plane indicates an antagonism. For each combination, three independent experiments were carried out to measure the dose–response curves of each individual compound and the combinations thereof.

## Results

### Novel Analogues of ART with Improved in vitro anti-HCV Activity

**Figure 3:**
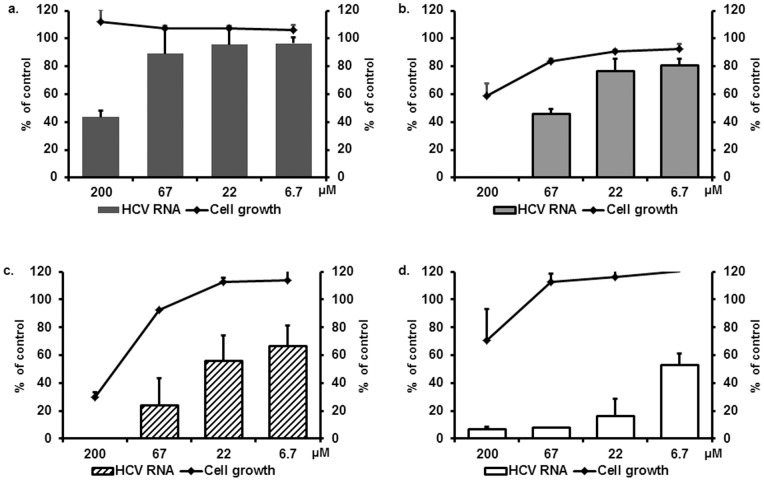
*In vitro* anti-HCV activity of Artemisinin and its selected analogues on the replication of infectious HCVcc as measured by means of qRT-PCR (n = 4). a) ART; b) TVN4; c) AJ-002 and d) AJ-004. Bars indicate the HCV RNA level as compared to control (%) and lines represent the cell growth as compared to untreated controls (%).

ART inhibits, as we demonstrated earlier, the *in vitro* replication of HCV subgenomic replicons (genotype 1b) in a selective and dose-dependent manner [27]. Here, we studied whether ART is also effective in hepatoma cells infected with the infectious HCV JFH-1. ART was found to inhibit HCV replication in a dose-dependent manner with EC_50_ value of 167±38 µM. At the highest concentration tested (400 µM), the host cell proliferation and cell viability were not affected (Fig. 3 and Table S1). Well known derivatives of ART such as Artesunate (ARS), Artemether (ARM) and Dihydroartemisinin (DHA) were found to be highly toxic in our hepatoma cell cultures (CC_50_<6 µM).

We next assessed the antiviral activity of novel ART derivatives (all were recently synthesized with the aim to improve the anti-malarial properties of this class of drugs [20]) belonging to three different categories (AJ, TVN and DW). The chemical structures of ART and the compounds of category AJ are depicted in Figure 1. All compounds of this group were found to be more active against HCV-cell culture (HCVcc) than ART (Fig. 3, Table S1). The EC_50_ values were 26±5, 15±2, and 16±4 µM for AJ-001, AJ-002 and AJ-004, respectively. The antiviral effect of these analogues was next assessed in the subgenomic HCV replicon system (Huh 5-2). The compounds exert anti-HCV activity against the genotype (1b) subgenomic replicon with AJ-001 and AJ-004 being the most potent with EC_50_ values of 8.8±2.7 and 3.2±2.4 µM, respectively (Table 1). Category TVN consists of 3 analogues (Fig. 2), of which TVN4 inhibits the replication of the infectious HCV at EC_50_ = 59±6 µM (Fig. 3) while inhibiting the subgenomic replicon at EC_50_ = 36±16 µM (Table 1). TVN2 and TVN6 had weak activities against the infectious HCV JFH (>70 µM). Of the 30 compounds of category DW (Figure S1), only one, i.e. DW 13, exerts anti-HCV activity at non-toxic concentrations in the HCVcc system and the subgenomic replicon assay (EC_50_-value ∼30 µM). All DW 13 related analogues proved toxic to the cells at ∼10 µM (Figure S1).

**Table 1:** Effect of ART and derivatives on Huh 5-2 HCV replicon replication.

Compound	EC_50_(µM)	CC_50_(µM)	+Hemin(5 µM)	+L-NAC (5 mM)
**ART**	75±7	>400	9.3±0.9 **(8)**	>400 **(−5)**
**AJ-001**	8.8±2.7	>133	4.6±2.8 **(2)**	26±2 **(−3)**
**AJ-002**	30±8	>133	1.9±0.7 **(15)**	68±22 **(−2)**
**AJ-004**	3.2±2.4	>133	4.0±0.1 **(0)**	17±4 **(−5)**
**TVN2**	25±13	36±7	6.3±2 **(+4)**	n.d
**TVN4**	36±16	123±14	17±6 **(+2)**	>100 **(−3)**
**TVN6**	3.6±2.3	40±20	n.d	n.d

∶ 50% effective concentration, CC50∶ 50% cytostatic concentration. Data obtained from the measurement of the firefly luciferase activity, and are mean values ± SD for four independent experiments (expressed in µM). EC50

µM, Hemin inhibits HCV replicon replication by 30%. Values between brackets indicate fold-change. At 5

### Hemin Potentiates the Anti-HCV Activity of ART and Derivatives

The malaria parasite is enriched in Hemin which results from the digestion and degradation of haemoglobin. Hemin was demonstrated to exert its anti-malarial activity, in part, by binding to the ART molecule forming Hemin-ART adduct from which radicals are released [28]. Hemin alone inhibits the replication of the HCV infectious virus in a dose dependent manner as measured by means of qRT-PCR (EC_50_ = 8.0±0.6 µM) and is not toxic to Huh 7.5.1 at concentrations >50 µM. At 5 µM, Hemin potentiates the antiviral activity of ART in the HCVcc system by a factor 2-fold and in the replicon model by a factor 8. The anti-HCV activity (in the subgenomic replicon system) of AJ-002, but not of AJ-004, was potentiated 15-fold by Hemin (Table 1). The combined treatment with Hemin was selective and did not increase the toxicity profile at the concentrations tested.

### Carbon-centered Radicals are not Crucial for the in vitro Anti-HCV Activity for ART and Analogues in Cultures

Formation of carbon-centered radicals has been reported to be critical for the *in vitro* anti-malarial activity of Artemisinin [29]. To study whether these radicals are or are not required for the anti-HCV activity of ART and its analogues, we combined a nitroxide radical spin trap, 2,2,6,6-tetramethyl-1-piperidinyloxy (TEMPO) compound with either ART or TVN4 in the HCV replicon system. TEMPO alone had no effect on the replication of HCV at concentrations up to 200 µM. The combination of ART or TVN4 with TEMPO resulted only in a marginaly antagonistic effect (Figure S2).

### ART and its most Potent Analogues Partially Inhibit the in vitro Replication of HCV by Induction of Reactive Oxygen Species (ROS)

A possible mechanism by which ART and analogues may exert their activity may be by the induction of reactive oxygen species (ROS) [30]. If so, the addition of an anti-oxidant should reduce their anti-HCV activity. We therefore combined in the HCV subgenomic replicon (Huh 5-2) assay ART with L-N-acetylcysteine (L-NAC); a compound that reduces reactive oxygen species (ROS) formation. Whereas L-NAC alone has no effect on the HCVcc replication at the concentration tested (HCV RNA replication was 98%±11 of UTC), the anti-HCV activity of ART and its analogues (TVN4, AJ-001, AJ-002 and AJ-004) was reduced by a factor 2 to 5 following the addition of L-NAC (Table 1, Figure S3).

## Discussion

Artemisinin (ART), a natural product isolated from the plant *Atremesia annua L*, was originally discovered during the Vietnam War as a potent treatment for malaria [10]. Besides its anti-malarial properties, ART also exerts *in vitro* anti-bacterial, anti-inflammatory and anti-angiogenic activity [15], [27], [31]. ART also inhibits the *in vitro* replication of the human cytomegalovirus (HCMV) and the hepatitis B virus (HBV) [10], [16], [32] and its derivative Artesunate inhibits the *in vitro* HIV replication [33]. We demonstrated previously that ART inhibits the *in vitro* HCV replicon replication [27]. Here, we report that ART inhibits also the replication of infectious HCV JFH-1.

ART derivatives such as Artesunate, Artemether and Dihydroartemisinin [34]–[37], that are currently being used to treat malarial infections, proved in our hands highly toxic in hepatoma cell cultures (Huh 7.5.1) and were not considered for further study. Interestingly, we were able to identify analogues that proved markedly more potent as HCV inhibitors than the parent compound.

In the subgenomic replicon system, the ART dimer (AJ-004) was to be about ∼3-fold more efficient in inhibiting the *in vitro* HCV replicon replication as compared to the monomer AJ-001, and was ∼10-fold more potent than the monomer AJ-002 (benzyl aldehyde derivative of ART). In cells infected with HCV JFH-1, the ART monomers were roughly equipotent to the dimer. This may suggest that the antiviral activity in the HCVcc-infected cells may be determined by properties other than those related to the endoperoxide bridge.

The malaria parasite has a high content of Hemin as a result of the haemoglobin digestion and degradation. Thus, Hemin may play a critical role in the anti-malarial activity of ART. It was suggested that the iron centre of Hemin attacks the endoperoxide bridge of the trioxane resulting in the cleavage of C3–C4 and the release of radicals. Iron binds O1 (not O2) of ART to form an iron-O-C bond (a Hemin-ART adduct) responsible for the biological activity of the compound [38]. Surprisingly, Hemin did not potentiate the anti-HCV activity of the most potent derivative (AJ-004). In line with previous findings [39]–[41], we showed that Hemin itself was able to inhibit the HCVcc replication. It is thus possible that the role of iron in the anti-HCV activity of ART and its derivatives may vary with the chemical structure of the compound.

Based on the observations made for the effect of the combination of ART (as well as the analogue TVN4) with a nitroxide radical spin trap (TEMPO) on anti-HCV activity, it is unlikely that carbon centred radicals are as important for the anti-HCV activity of ART as was suggested for the anti-malarial activity of the compound.

The cleavage of the endoperoxide bridge within the ART molecule results in the release of carbon radicals and reactive oxygen species (ROS). The induction of ROS has been demonstrated to regulate the replication of other viruses such as HBV (negatively) [42] or HIV (positively) [43]. For HCV, it was shown that peroxide treatment (which results in ROS induction), at concentrations that were not toxic to the cells, resulted in the disruption of active HCV replication complexes through reduction of the amount of NS3 and NS5A in the replication complexes [42]. The anti-HCV activity of ART induced by peroxides could be negated by L-N-Acetylcysteine (L-NAC) [the molecule that inhibits ROS generation]. Therefore, we studied the anti-HCV activity of ART or analogues in combination with L-N-Acetylcysteine (L-NAC). L-NAC reduced the anti-HCV activity of ART and derivatives (2 to 5 fold) (Table 1).

In conclusion, we identified novel derivatives of ART that are markedly more potent and selective *in vitro* HCV inhibitors than the parent compound. It is suggested that at least part of the antiviral activity is related to the induction of ROS. Carbon-centred radicals are only marginally involved in the anti-HCV activity of ART and derivatives thereof.

## Supporting Information

Figure S1 **Structural formulae of Artemisinin and synthetic derivatives belonging to the third category DW.**
(DOC)

Figure S2 **Combination studies of ART and TVN4 with TEMPO in Huh 5-2 cells: zero plane indicates to additive effect on the z-axis, while all values above zero point to a synergistic effect, and all values below zero indicate an antagonistic effect.**
(DOC)

Figure S3 ***In vitro*** **anti-HCV subgenomic replicon activity (in Huh-5-2) of a. ART, b. AJ-001, c. AJ-002 and d. AJ-004 in combination with hemin or L-NAC.**
(DOC)

Table S1 Effect of ART and its analogues on the replication of HCVcc.
(DOC)
