# Supplementary material for: Artemisinin Analogues as Potent Inhibitors of In Vitro Hepatitis C Virus Replication
Source: PLoS One. 2013 Dec 11;8(12):e81783. doi: 10.1371/journal.pone.0081783 (PMC3859510; doi:10.1371/journal.pone.0081783)
Supplement: Figure S1 — Structural formulae of Artemisinin and synthetic derivatives belonging to the third category DW. (DOC) [file pone.0081783.s001.doc]

**Figure S1.** Structural formulae of Artemisinin and synthetic derivatives belonging to the third category DW.
