# Supplementary material for: Artemisinin Analogues as Potent Inhibitors of In Vitro Hepatitis C Virus Replication
Source: PLoS One. 2013 Dec 11;8(12):e81783. doi: 10.1371/journal.pone.0081783 (PMC3859510; doi:10.1371/journal.pone.0081783)
Supplement: Figure S2 — Combination studies of ART and TVN4 with TEMPO in Huh 5-2 cells: zero plane indicates to additive effect on the z-axis, while all values above zero point to a synergistic effect, and all values below zero indicate an antagonistic effect. (DOC) [file pone.0081783.s002.doc]

**Figure S2. Combination studies of ART and TVN4 with TEMPO in Huh 5-2 cells:** zero plane indicates to additive effect on the z-axis, while all values above zero point to a synergistic effect, and all values below zero indicate an antagonistic effect.
