# Supplementary material for: Artemisinin Analogues as Potent Inhibitors of In Vitro Hepatitis C Virus Replication
Source: PLoS One. 2013 Dec 11;8(12):e81783. doi: 10.1371/journal.pone.0081783 (PMC3859510; doi:10.1371/journal.pone.0081783)
Supplement: Figure S3 — In vitro anti-HCV subgenomic replicon activity (in Huh-5-2) of a. ART, b. AJ-001, c. AJ-002 and d. AJ-004 in combination with hemin or L-NAC. (DOC) [file pone.0081783.s003.doc]

**a.**

**b.**

**c.**

**d.**

**Figure S3**. *In vitro* anti-HCV subgenomic replicon activity (in Huh-5-2) of a. ART, b. AJ-001, c. AJ-002 and d. AJ-004 in combination with hemin or L-NAC.
