# Supplementary material for: Artemisinin Analogues as Potent Inhibitors of In Vitro Hepatitis C Virus Replication
Source: PLoS One. 2013 Dec 11;8(12):e81783. doi: 10.1371/journal.pone.0081783 (PMC3859510; doi:10.1371/journal.pone.0081783)
Supplement: Table S1 — Effect of ART and its analogues on the replication of HCVcc. (DOC) [file pone.0081783.s004.doc]

| **Table S1.** Effect of ART and its analogues on the replication of HCVcc. | | | | |
| --- | --- | --- | --- | --- |
| **Compound** | **EC_50_ (µM)** | **CC_50_ (µM)** | **SI** | |
| **ART** | 167 ± 38 | > 400 | > 2 | |
| **AJ-001** | 26 ± 5 | > 133 | > 5 | |
| **AJ-002** | 15 ± 2 | > 133 | > 8 | |
| **AJ-004** | 16 ± 4 | > 133 | > 8 | |
| **TVN4** | 52 ± 6 | > 133 | > 2 | |
| **DW13** | 31 ± 11 | > 133 | > 4 | |
| **VX-950** | 0.2 ± 0.1 | > 10 | > 50 | |
| EC_50_: 50% effective concentration, CC_50_: 50% cytostatic concentration. Data are expressed in μM and are mean values ± SD for four independent experiments. | | | |  |
